# An evaluation of linear and non-linear models of expressive dynamics in classical piano and symphonic music

**DOI:** 10.1007/s10994-017-5631-y

**Published:** 2017-03-09

**Authors:** Carlos Eduardo Cancino-Chacón, Thassilo Gadermaier, Gerhard Widmer, Maarten Grachten

**Affiliations:** 1 Austrian Research Institute for Artificial Intelligence, Vienna, Austria; 2 Department of Computational Perception, Johannes Kepler University, Linz, Austria

**Keywords:** Musical expression, Non-linear basis models, Artificial neural networks, Computational models of music performance

## Abstract

Expressive interpretation forms an important but complex aspect of music, particularly in Western classical music. Modeling the relation between musical expression and structural aspects of the score being performed is an ongoing line of research. Prior work has shown that some simple numerical descriptors of the score (capturing dynamics annotations and pitch) are effective for predicting expressive dynamics in classical piano performances. Nevertheless, the features have only been tested in a very simple linear regression model. In this work, we explore the potential of non-linear and temporal modeling of expressive dynamics. Using a set of descriptors that capture different types of structure in the musical score, we compare linear and different non-linear models in a large-scale evaluation on three different corpora, involving both piano and orchestral music. To the best of our knowledge, this is the first study where models of musical expression are evaluated on both types of music. We show that, in addition to being more accurate, non-linear models describe interactions between numerical descriptors that linear models do not.

## Introduction

Performances of written music by humans are hardly ever exact acoustical renderings of the notes in the score, as a computer would produce. Nor are they expected to be: a natural human performance involves an interpretation of the music, in terms of structure, but also in terms of affective content (Clarke 1988; Palmer 1997), which is conveyed to the listener by local variations in tempo and loudness, and, depending on the expressive possibilities of the instrument, the timing, articulation, and timbre of individual notes.

Musical expression is a complex phenomenon. Becoming an expert musician takes many years of training and practice, and rather than adhering to explicit rules, achieved performance skills are to a large degree the effect of implicit, procedural knowledge. This is not to say that regularities cannot be found in the way musicians perform music. Decades of empirical research have identified a number of factors that jointly determine the way a musical piece is rendered (Palmer 1996; Gabrielsson 2003). For example, aspects such as phrasing (Todd 1992), meter (Sloboda 1983), but also intended emotions (Juslin 2001), all have an effect on expressive variations in music performances.

A better understanding of musical expression is not only desirable in its own right, as scientific knowledge. The potential role of computers in music creation will also depend on accurate computational models of musical expression. For example, music software such as MIDI sequencers and music notation editors may benefit from such models in that they enable automatic or semi-automatic expressive renderings of musical scores.

Several methodologies have been used to study musical expression, each with their own merits. Considerable contributions to our current knowledge on musical expression have been made by works following the paradigm of experimental psychology, in which controlled experiments are designed and executed, investigating a single aspect of performance, such as the timing of grace notes (Timmers et al. 2002), or cyclic rhythms (Repp et al. 2013). Local variations in tempo as a function of phrasing have also been explicitly addressed, using computational models (Todd 1992; Friberg and Sundberg 1999). Complementary to such approaches, often testing a specific hypothesis about a particular aspect of expression, data mining and machine learning paradigms set out to discover regularities in musical expression using data sets comprising musical performances (Widmer 2003; Ramirez and Hazan 2004). Given the implicit nature of expressive performance skills, an advantage of the latter approach is that it may reveal patterns that have gone as of yet unnoticed, perhaps because they do not relate in any obvious way to existing scholarly knowledge about expressive performance, or even because they are so self-evident to experts that they escape attention.

A computational framework has been proposed in Grachten and Widmer (2012), to model the effect of structural aspects of a musical score on expressive performances of that score, in particular expressive dynamics (the relative intensity with which the notes are performed). This framework, referred to as the basis function modeling (BM) approach, follows the machine learning paradigm in that it estimates the parameters of a model from a set of recorded music performances, for which expressive parameters such as local loudness, tempo, or articulation, can be measured or computed.

The essential characteristic of BM is its use of *basis functions* as a way to describe structural properties of a musical score, ranging from the metrical position of the notes, to the presence and scope of certain performance directives. For instance, a basis function for the performance directive ***forte*** (***f***), may assign a value of 1 to notes that lie within the scope of the directive, and 0 to notes outside the scope. Another basis function may assign a value of 1 to all notes that fall on the first beat of a measure, and 0 to all other notes. But basis functions are not restricted to act as indicator functions; They can be any function that maps notes in a score to real values. For example, a useful basis function proves to be the function that maps notes to (powers of) their MIDI pitch values. Given a set of such basis functions, each representing a different aspect of the score, the intensity of notes in an expressive performance is modeled simply as a linear combination of the basis functions. The resulting model has been used for both predictive and analytical purposes (Grachten and Widmer 2012; Grachten et al. 2014).

The original formulation of the BM approach, referred to as the linear basis models (LBMs), used a least squares (LS) regression to compute the optimal model parameters. Subsequently, a probabilistic version of the LBMs using Bayesian linear regression was presented in Grachten et al. (2014), where the prior distribution of the model parameters was assumed to be a zero-mean isotropic Gaussian distribution. This probabilistic version was expanded to Gaussian priors with arbitrary mean and covariance in Cancino Chacón et al. (2014).

Although the linear model produces surprisingly good results given its simplicity, a question that has not generally been answered is whether the same basis function framework can benefit from a more powerful, non-linear model. Rather than score properties in isolation, it is conceivable that expressive variations depend on *interactions* between score properties. Moreover, musical expression may depend on score properties in ways that are not well-approximated by a linear relation. Therefore, in this paper, we propose two neural network based non-linear basis models (NBMs). Although there are many ways to model non-linear relationships, artificial neural network (ANNs) modeling offers a flexible and conceptually simple approach, that has proven its merits over the past decades.

Thus, the purpose of this paper is to investigate whether the basis function modeling approach to expressive dynamics benefits from non-linear connections between the basis functions and the targets to be modeled.

To this end, we run a comparison of the LBM and the NBM approaches on two data sets of professional piano performances of Chopin piano music and Beethoven piano sonatas, and a data set of orchestral performances of Symphonies from the classical and romantic periods. Apart from the predictive accuracy of both models, we present a (preliminary) qualitative interpretation of the results, by way of a sensitivity analysis of the models.

The outline of this paper is as follows: in Sect. 2, we discuss prior work on computational models of musical expression. In Sect. 3, the basis function modeling approach for musical expression is presented in some more detail. A mathematical formulation of the presented models is provided in Sect. 4. In Sect. 5, we describe the experimental comparison mentioned above. The results of this experimentation are presented and discussed in Sect. 6. Conclusions are presented in Sect. 7.

## Related work

Musical performance represents an ongoing research subject that involves a wide diversity of scientific and artistic disciplines. On the one hand, there is an interest in understanding the cognitive principles that determine the way a musical piece is performed (Clarke 1988; Palmer 1997) such as the effects of musical imagery in the anticipation and monitoring of the performance of musical dynamics (Bishop et al. 2014). On the other hand, computational models of expressive music performance attempt to investigate the relationships between certain properties of the musical score and performance context with the actual performance of the score (Widmer and Goebl 2004). These models can serve mainly analytical purposes (Widmer 2002; Windsor and Clarke 1997), mainly generative purposes (Teramura et al. 2008), or both (Grindlay and Helmbold 2006; De Poli et al. 2001; Grachten and Widmer 2012).

Computational models of music performance tend to follow two basic paradigms: *rule based* approaches, where the models are defined through music-theoretically informed rules that intend to map structural aspects of a music score to quantitative parameters that describe the performance of a musical piece, and *data-driven* (or *machine learning*) approaches, where the models try to infer the rules of performance from analyzing patterns obtained from (large) data sets of observed (expert) performances (Widmer 2003).

One of the most well-known rule-based systems for musical music performance was developed at the Royal Institute of Technology in Stockholm (referred to as the KTH model) (Friberg et al. 2006). This system is a top-down approach that describes expressive performances using a set of carefully designed and tuned performance rules that predict aspects of timing, dynamics and articulation, based on a local musical context.

Among the machine learning methods for musical expression is the model proposed by Bresin (1998). This model uses artificial neural networks (NNs) in a supervised fashion in two different contexts: (1) to learn and predict the rules proposed by the KTH model and (2) to learn the performing style of a professional pianist using an encoding of the KTH rules as inputs. Although similar in spirit, the NBM proposed in this paper uses a lower level representation of the score, and makes less assumptions on how the different score descriptors contribute to the expressive dynamics.

Grachten and Krebs (2014) and van Herwaarden et al. (2014) use unsupervised feature learning as the basis for modeling expressive dynamics. These approaches learn features describing the local context of each note in a score from a piano roll representation of such score. These features are then combined in a linear model, which is trained in a supervised fashion using LS to predict the loudness of each note in a performance. Although the unsupervised learning of representations of the musical score is clearly of interest in the discovery of knowledge about musical expression, a drawback is that the piano roll encoding of the musical score does not include performance directives written by the composer, such as dynamics or articulation markings (such as ***piano***, staccato, etc), nor potentially relevant aspects like the metrical position of notes, or slurs. Both the KTH system and previous work on LBMs have shown that the encoding of dynamics/articulation markings plays an important role in the rendering of expressive performances.

A broader overview of computational models of expressive music performance can be found in Widmer and Goebl (2004).

## The basis function model of expressive dynamics

In this section, we describe the basis function modeling (BM) approach, independent of the linear/non-linear nature of the connections to the expressive parameters. First we introduce the approach as it has been used to model expressive dynamics in solo piano performances. After that, we briefly describe some extensions that are necessary to accommodate for modeling loudness measured from recorded ensemble performances, as proposed in Gadermaier et al. (2016).

As mentioned above, musical expression can be manifested by a variety of facets of a performance, depending on the instrument. The BM approach described here can be used to model expressive variations in different dimensions, and accordingly, work on modeling local tempo (Grachten and Cancino Chacón 2017), and joint modeling of different performance aspects is ongoing. In the present study however, we focus on expressive dynamics, or local variations in loudness over the course of a performance.

### Modeling expressive dynamics in solo piano performances

We consider a *musical score* a sequence of elements (Grachten and Widmer 2012). These elements include note elements (e.g. pitch, duration) and non-note elements (e.g. dynamics and articulation markings). The set of all note elements in a score is denoted by $\mathcal {X}$. Musical scores can be described in terms of *basis functions*, i.e. numeric descriptors that represent aspects of the score. Formally, we can define a basis function $\varphi$ as a real valued mapping $\varphi {:}\,\mathcal {X} \mapsto \mathbb {R}$. The expressive dynamics of the performance are conveyed by the *MIDI velocities* of the performed notes, as recorded by the instrument (see Sect. 5.1). By defining basis functions as functions of notes, instead of functions of time, the BM framework allows for modeling forms of music expression related to simultaneity of musical events, like the micro-timing deviations of note onsets in a chord, or the melody lead (Goebl 2001), i.e. the accentuation of the melody voice with respect to the accompanying voices by playing it louder and slightly earlier.

**Fig. 1:**
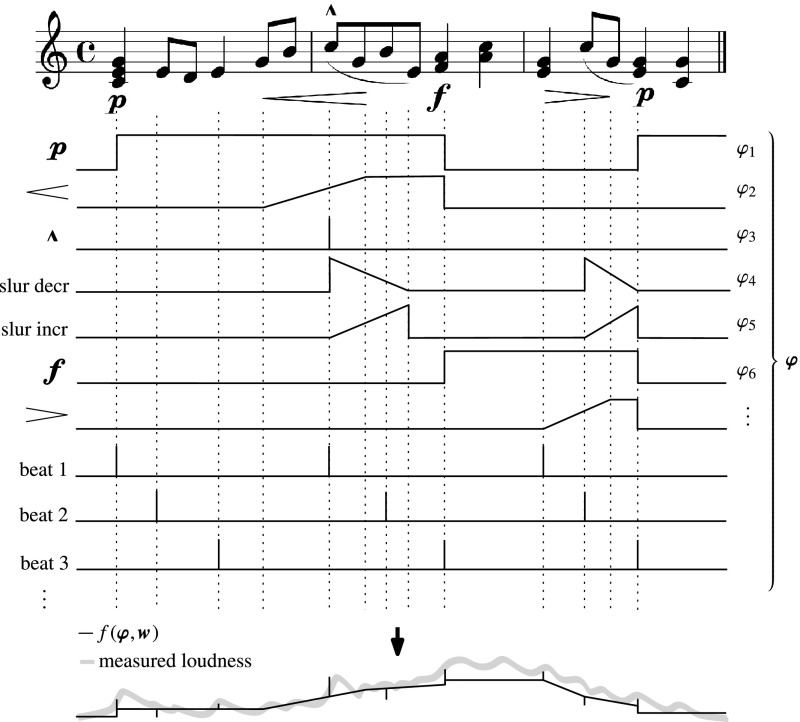
Schematic view of expressive dynamics as a function $f(\varvec{\varphi },\mathbf {w})$ of basis functions $\varphi$, representing dynamic annotations

Figure 1 illustrates the idea of modeling expressive dynamics using basis functions schematically. Although basis functions can be used to represent arbitrary properties of the musical score (see Sect. 3.2), the BM framework was proposed with the specific aim of modeling the effect of *dynamics markings*. Such markings are hints in the musical score, to play a passage with a particular dynamical character. For example, a ***p*** (for ***piano***) tells the performer to play a particular passage softly, whereas a passage marked ***f*** (for ***forte***) should be performed loudly. Such markings, which specify a constant loudness that lasts until another such directive occurs, are modeled using a step-like function, as shown in the figure. Another class of dynamics markings, such as ***marcato*** (i.e. the “hat” sign over a note), or textual markings like ***sforzato*** (***sfz***), or ***forte piano*** (***fp***), indicate the accentuation that note (or chord). This class of markings is represented through (translated) unit impulse functions. Gradual increase/decrease of loudness (***crescendo***/***diminuendo***) is indicated by right/left-oriented wedges, respectively. Such markings form a third class, and are encoded by ramp-like functions. Note that the effect of a ***crescendo***/***diminuendo*** is typically persistent, in the sense that the increase/decrease of loudness is not “reset” to the initial level at the end of the ***crescendo***/***diminuendo***. Rather, the effect persists until a new constant loudness directive occurs. Furthermore, note that although the expected effect of a ***diminuendo*** is a decrease in loudness, the basis function encoding of a ***diminuendo*** is an increasing ramp. The magnitude and sign of the effect of the basis function on the loudness are to be inferred from the data by the model.

In the BM approach, the expressive dynamics (i.e. the MIDI velocities of performed notes) are modeled as a combination of the basis functions, as displayed in the figure.

### Groups of basis functions

As stated above, the BM approach encodes a musical score into a set of numeric descriptors. In the following, we describe various groups of basis functions, where each group represents a different aspect of the musical score. The information conveyed by the basis functions is either explicitly available in the score (such as dynamics markings, and pitch), or can be inferred from it in a straight-forward manner (such as metrical position, and interonset-intervals).

1. *Dynamics markings* Bases that encode dynamics markings, such as shown in Fig. 1. Basis functions that describe gradual changes in loudness, such as ***crescendo*** and ***diminuendo***, are represented through a combination of a ramp function, followed by a constant (step) function, that continues until a new constant dynamics marking (e.g. ***f***) appears, as illustrated by $\varphi _2$ in Fig. 1.
2. *Pitch* A basis function that encodes the pitch of each note as a numerical value. More specifically, this basis function simply returns the MIDI note value of the pitch of a note, normalized to the range [0, 1].
3. *Vertical neighbors* Two basis functions that evaluate to the number of simultaneous notes with lower, and higher pitches, respectively, and a third basis function that evaluates to the total number of simultaneous notes at that position.
4. *IOI* The inter-onset-interval (IOI) is the time between the onsets of successive notes. For note *i*, a total of six basis functions represent the IOIs between the three previous onsets and the next three onsets, i.e., the onsets between $(i-2,i-3)$, $(i-1,i-2)$, $(i, i-1)$, $(i, i+1)$, $(i+1,i+2)$, and $(i+2,i+3)$. These basis functions provide some context of the (local) rhythmical structure of the music.
5. *Ritardando* Encoding of markings that indicate gradual changes in the tempo of the music; Includes functions for ***rallentando***, ***ritardando***, ***accelerando***.
6. *Slur* A representation of *legato* articulations indicating that musical notes are performed smoothly and connected, i.e. without silence between each note. The beginning and ending of a slur are represented by decreasing and increasing ramp functions, respectively. The first (denoted *slur decr*) ranges from one to zero, while the second (denoted *slur incr*) ranges from zero to one over the course of the slur.
7. *Duration* A basis function that encodes the duration of a note. While the IOI describes the time interval between two notes, the duration of a note refers to the time that such note is sounding. In piano music, particularly, the duration of a note describes the time between a key is pressed and released, while the IOI describes the time between pressing two keys.
8. *Rest* Indicates whether notes precede a rest.
9. *Metrical* Representation of the time signature of a piece, and the (metrical) position of each note in the bar. For each time signature , there are $a + 1$ basis functions: *a* basis functions indicate notes starting at each beat and a single basis function indicates notes starting on a *weak* metrical position. For example, the basis function labeled  evaluates to 1 for all notes that start on the first beat in a  time signature, and to 0 otherwise. Although in (western) music, most time signatures have common accentuation patterns, we choose not to hard-code these accentuation patterns in the form of basis functions. Instead, by defining the metrical basis functions as sets of indicator functions associated to metrical positions, we leave it to the basis models to *learn* the accentuation patterns as they occur in the data.
10. *Repeat* Takes into account repeat and ending barlines, i.e. explicit markings that indicate the structure of a piece by indicating the end of a particular section (which can be repeated), or the ending of a piece. The barlines are represented by an anticipating ramp function leading up to the repeat/ending barline over the course of a measure.
11. *Accent* Accents of individual notes or chords, such as the ***marcato*** in Fig. 1.
12. *Staccato* Encodes ***staccato*** markings on a note, an articulation indicating that a note should be temporally isolated from its successor, by shortening its duration.
13. *Grace notes* Encoding of musical ornaments that are melodically and or harmonically nonessential, but have an embellishment purpose.
14. *Fermata* A basis function that encodes markings that indicate that a note should be prolonged beyond its normal duration.
15. *Harmonic* Two sets of indicator basis functions that encode a computer-generated harmonic analysis of the score based on the probabilistic polyphonic key identification algorithm proposed in Temperley (2007). This harmonic analysis produces an estimate of the key and scale degree, i.e. the roman numeral functional analysis of the harmony of the piece, for each bar of the score. A set of basis functions encode all major and minor keys while another set of basis functions encodes scale degrees.

Note that while some basis functions, notably those that encode dynamics markings, are semantically connected to the expressive dynamics, others are not. In particular, ***ritardando*** and ***fermata*** markings are semantically related to *tempo*, rather than *dynamics*. Nevertheless, we choose to include information about this kind of markings, since they tend to coincide with the endings of phrases or other structural units, which are likely to be relevant for expressive dynamics, but not explicitly encoded in the musical score.

It should be noted that some of the above mentioned groups of basis functions can describe a large number of individual basis functions. For example, the traditional binary and ternary time signatures (encoded in the group of Metrical basis functions) generate more than 50 basis functions with the  time signature alone generating 13 basis functions, namely , and .

### From solo to ensemble performance

The BM framework described above assumes that a loudness value is present for each note in the piece. For piano performances recorded on a special computer-monitored piano (see Sect. 5.1) this is the case. In such recordings, the recorded *hammer velocity* of the piano keys is a direct representation of the dynamics, revealing how loud each individual note is played. For acoustic instruments other than the piano, such precise recording techniques are not available, and therefore the dynamics of music ensembles such as symphonic orchestras cannot be measured in a similar way. Another approach would be to record each instrument of the orchestra separately, and measure the loudness variations in each of the instruments, but this approach is not feasible either, since apart from the financial and practical barriers, the live setting in which orchestras normally play prevents a clean separation of the recording channels by instrument. This means we are left with only a rudimentary representation of dynamics, namely the overall variation of loudness over time, measured from the orchestral recording.

The way dynamics is measured and represented has repercussions for the basis function modeling approach. In contrast to the digital grand piano setting, the overall loudness measured from an orchestral recording does not provide a loudness value for each performed note, but one per time instant. Thus, basis function information describing multiple simultaneous notes must be combined to account for a single loudness value. We do so by defining *fusion* operators for subsets of basis functions. In most cases, the *average* operator is an adequate form of fusing basis function information. For some basis functions however, we use the *sum* operator, in order to preserve information about the number of instances that were fused into a single instrument. Future experimentation should provide more informed choices as to the optimal fusion operators to use, including the use of different weighting strategies and nonlinear aggregation of the basis functions.

**Fig. 2:**
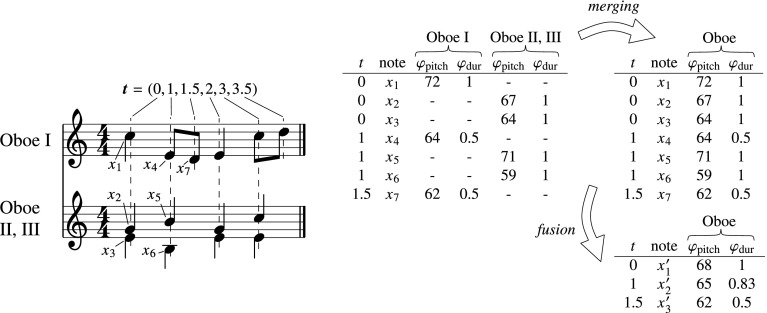
Illustration of *merging* and *fusion* of score information of two different parts belonging to the same instrument class “Oboe”. The matrix on the *left* shows two example basis functions, $\varphi _{\mathrm {pitch}}$ and $\varphi _{\mathrm {dur}}$, for the first notes of each of the two score parts. The matrix *top right* is the result of *merging* basis functions of different “Oboe” instantiations into a single set. The matrix on the *bottom right* is the result of *fusion*, applied per basis function to each set of values occurring at the same time point

Another significant change with respect to the solo instrument setting is that in an ensemble setting, multiple sets of basis functions are produced, each set describing the score part of a particular instrument. In a symphonic piece, multiple instantiations of the same instrument may be present. Moreover, different pieces may have different instrumentations. This poses a challenge to an expression model, which should account for the influence of instruments consistently from one piece to the other. We address this issue by defining a *merging* operation that combines the information of different sets of basis functions for each instance of an instrument into a single set of basis functions per instrument class. Both the merging and the fusion operations are illustrated for a musical excerpt in Fig. 2.

The proposed description of orchestral music can easily generate a very large amount of basis functions, since each instrument has its own set of basis functions. For example, the metrical basis functions corresponding to the traditional binary and ternary time signatures for the classical string section^1^ generate more than 250 basis functions (e.g., , etc.).

## Linear, non-linear, and recurrent non-linear basis models

In the previous section we have described how the musical scores of solo and ensemble pieces can be described by means of basis functions, and how expressive dynamics can be measured, but we have not yet described how to model the relation between expressive dynamics and the basis functions. In this section we describe three models of increasing complexity.

In the following, $\mathcal {X}=\{x_1,\ldots ,x_N\}$ represents the set of *N* notes in a musical score. The values of the basis functions corresponding to note $x_i$ are denoted by a vector $\varvec{\varphi }(x_i)=(\varphi _1(x_i),\ldots ,\varphi _M(x_i))^{\mathrm {T}}\in \mathbb {R}^{M}$. In this way, we can represent a whole musical score as $\varvec{\varPhi } \in \mathbb {R}^{N \times M}$, a matrix with elements $\varPhi _{ij}=\varphi _j(x_i)$. The values of the expressive parameters for each note predicted by a BM model are represented by vector $\mathbf {y}=(y_1,\ldots ,y_N)^{\mathrm {T}}\in \mathbb {R}^N$. We can model the expressive parameters as a function of the input basis functions as

1 $$\begin{aligned} \mathbf {y}= f(\varvec{\varPhi }; \mathbf {w}), \end{aligned}$$

where $f(\cdot )$ is a function of $\varvec{\varPhi }$ given parameters $\mathbf {w}$.

### Linear basis models (LBMs)

The simplest way to explore the influence of the basis functions in the expressive parameters is using a linear regression. The LBM models expressive dynamics of the *i*th note in a score $x_i$ as a weighted sum of the basis functions as

2 $$\begin{aligned} y_{i} =\varvec{\varphi }(x_i)^{\mathrm {T}}\mathbf {w}, \end{aligned}$$

where $\mathbf {w}\in \mathbb {R}^{M}$ is a vector of weights.

### Non-linear basis models (NBM)

The influence of the basis functions in the expressive parameter can be modeled in a non-linear way using feed forward neural networks (FFNNs). These neural networks can be described as a series of (non-linear) transformations of the input data (Bishop 2006). Using this formalism, we can write the expressive parameter corresponding to the *i*th note as the output of a fully-connected FFNN with *L* *hidden layers* as

3 $$\begin{aligned} {y_i = f^{(L)} \left( \mathbf {w}^{(L)^{\mathrm {T}}}\mathbf {h}_i^{(L-1)} + w_0^{(L)} \right) ,} \end{aligned}$$

where $\mathbf {h}_i^{(L-1)}\in \mathbb {R}^{D_{L-1}}$ is the activation of the $(L-1)$th hidden layer (with $D_{L-1}$ units) corresponding to the *i*th note; $f^{(L)}(\cdot )$ is an element-wise activation function and $\mathbf {w}^{(L)}\in \mathbb {R}^{D_{L-1}}$ and $w_0^{(L)}\in \mathbb {R}$ are the vector of weights and a scalar bias of the *L*th layer, respectively. The activation for the *l*th hidden layer corresponding to the *i*th note $\mathbf {h}_i^{(l)}\in \mathbb {R}^{D_l}$, where $D_l$ is the number of units in the layer, is given by

4 $$\begin{aligned} \mathbf {h}_i^{(l)} = f^{(l)} \left( \mathbf {w}^{(l)} \mathbf {h}_i^{(l-1)} + \mathbf {w}_0^{(l)} \right) , \end{aligned}$$

where $\mathbf {w}^{(l)}\in \mathbb {R}^{D_l \times D_{l-1}}$ and $\mathbf {w}_0^{(l)}\in \mathbb {R}^{D_l}$ are the matrix of weights and the bias vector of the *l*th layer; and $\mathbf {h}_i^{(l-1)}\in \mathbb {R}^{D_{l-1}}$ is the activation of the $(l-1)$th hidden layer. The element-wise activation function of the *l*th layer is represented by $f^{(l)}$. As a convention, the 0th layer represents the input itself, i.e. $\mathbf {h}_i^{(0)} = \varvec{\varphi }(x_i)$. The set of all parameters is $\mathbf {w}=\{\mathbf {w}^{(1)}_0, \mathbf {w}^{(1)}, \ldots , w^{(L)}_0, \mathbf {w}^{(L)}\}$. Common activation functions for the hidden layers are sigmoid, hyperbolic tangent, softmax and rectifier ($ReLU(\xi ) = \max (0,\xi )$). Since we are using the FFNN in a regression scenario, the activation function of the last layer is set to the identity function ($f^{(L)}(\xi ) = \xi$) (Bishop 2006).

**Fig. 3:**
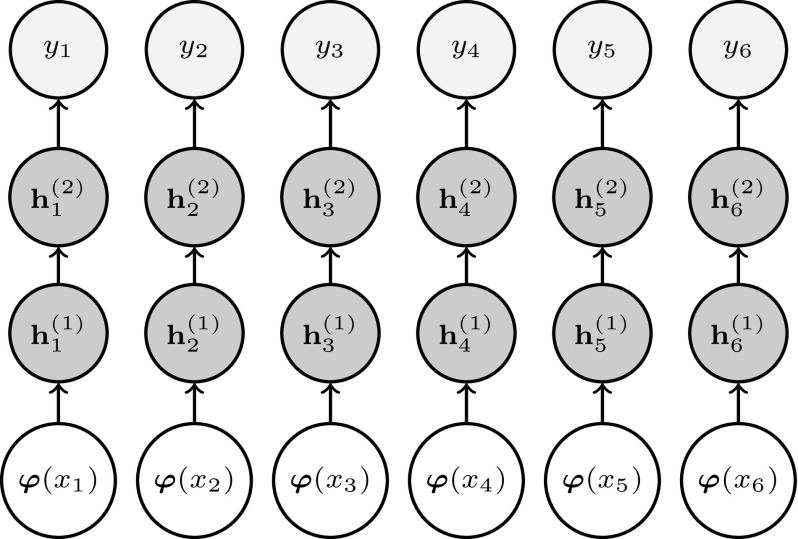
The architecture of the used NBM for modeling expressive dynamics. From *bottom* to *top*, the *circles* represent the input layer, two successive hidden layers and the output layer, respectively. From *left* to *right*, advancing time steps are shown

Figure 3 schematically illustrates an NBM with 2 hidden layers.

### Recurrent non-linear basis models (RNBMs)

Both the LBM and NBM models are static model that do not allow for modeling temporal dependencies within parameters. This problem can be addressed by using recurrent neural networks (RNNs). The basic structure of an RNN is the recurrent layer. The output of one such layer at time *t*, can be written as

5 $$\begin{aligned} \mathbf {h}_t = f_h\left( g_\varphi \left( \varvec{\varphi }(x_t)\right) + g_h \left( \mathbf {h}_{t^*}\right) \right) , \end{aligned}$$

where $g_\varphi \left( \varvec{\varphi }(x_t)\right)$ represents the contribution of the input of the network at time *t*, $g_h \left( \mathbf {h}_{t^*}\right)$ is the contribution of other time steps (past or future, or a combination of both) of the state of the recurrent layer. As in the case of NBMs, $f_h(\cdot )$ is an elementwise (non-linear) activation function. The output of the network can be computed in a similar fashion to traditional FFNNs using Eq. (3), where the non-recurrent and recurrent hidden layers are computed using Eqs. (4) and (5), respectively. For modeling expressive dynamics, we use Bidirectional RNNs (Schuster and Paliwal 1997). In this way, we combine information from the past and future score information to make a prediction of the dynamics of the current note.

While it is theoretically possible for large enough vanilla RNNs to predict sequences of arbitrary complexity, it has been shown that numerical limitations of training algorithms do not allow them to properly model long temporal dependencies (Pascanu et al. 2013). To address these problems, the Long Short Term Memory (LSTM) architecture were proposed in Hochreiter and Schmidhuber (1997). LSTMs include special purpose recurrent layers with memory cells that allow for a better finding and exploiting long range dependencies in the data. A more mathematical formulation of RNNs can be found in Graves (2013).

**Fig. 4:**
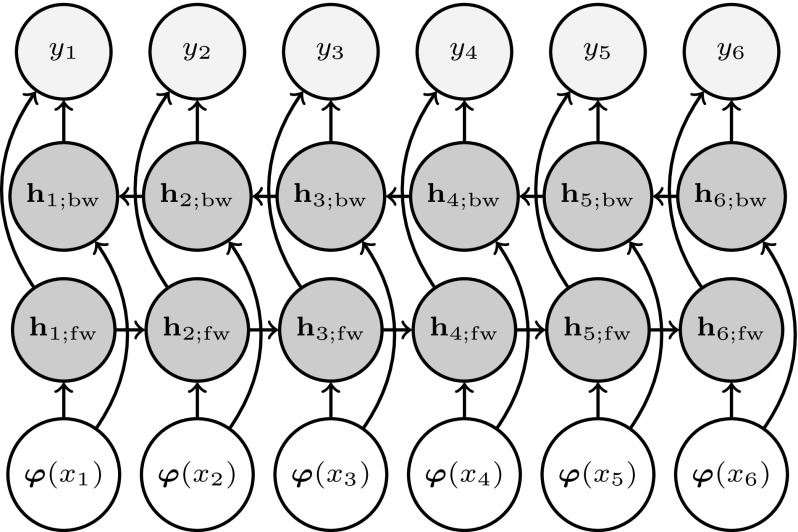
Bidirectional RNBM for modeling expressive dynamics. The single hidden layer $\mathbf {h}$ is made up from forward (fw) and backward (bw) recurrent hidden units. *Left* to *right* shows advancing time steps

Figure 4 shows the scheme of an RNBM with a single bidirectional layer.

### Learning the parameters of the model

Given $\varvec{\mathcal {T}}= \{ (\varvec{\varPhi }_1, \mathbf {t}_1), \ldots , (\varvec{\varPhi }_{K}, \mathbf {t}_K)\}$, a set of training pairs of scores and their corresponding expressive targets, the parameters of the models presented above can be estimated in a supervised way by minimizing the *mean squared error* between predictions and targets, i.e.

6 $$\begin{aligned} \hat{\mathbf {w}} = \mathop {{{\mathrm{\arg \!\min }}}}\limits _{\mathbf {w}} \frac{1}{K}\sum _k {||}{f(\varvec{\varPhi }_k; \mathbf {w}) - \mathbf {t}_k}{||}^2. \end{aligned}$$

### A note on evaluation strategies for computational models of musical expression

It is important to emphasize that the mean squared error (or other derived measures) with respect to a human performance is not necessarily a reliable indicator of the musical quality of a generated performance. Firstly, a low error does not always imply that a performance *sounds* good—at crucial places, a few small errors may make the performance sound severely awkward, even if these error hardly affect the overall error measure. Secondly, the very notion of expressive freedom implies that music can often be performed in a variety of ways that are very different, but equally convincing in their own right. In that sense, evaluating the models only by their ability to predict the performance of a single performer is not sufficient in the long run.

In spite of that, there are good reasons in favor of the mean squared error as a guide for evaluating performance model. Firstly, music performance is not *only* about individual expressive freedom. The works of Repp (e.g. Repp 1992, 1994) have shown that there are substantial commonalities in the variations of timing and dynamics across performers. We believe that assessing how well the model predicts existing human performances numerically does tell us something about the degree to which it has captured general performance principles. Secondly, model assessment involving human judgments of the perceptual validity of output is a time-consuming and costly effort, not practicable as a recurring part of an iterative model development/evaluation process. For such a process, numerical benchmarking against performance corpora is more appropriate. Note however that anecdotal perceptual validation of the BMs has taken place on several occasions, in the form of concerts/competitions where the BM, along with competing models was used to perform musical pieces live in front of an audience, on a computer-controlled grand piano.^2^

## Experiments

To determine to what degree the different model types are able to account for expressive dynamics, we subject them to a comparative evaluation on different data sets. For each of three data sets, the accuracy of the model predictions are tested using fivefold cross validation, where the set of instances belonging to a single musical piece was either entirely part of the training set, the validation set, or the test set, respectively. This is important since otherwise, repeated structures in the music that end up in different sets may cause an overly optimistic impression of the generalizing capabilities of the models.

### Data sets

Below, we describe the three different data sets used. The first two consist of classical solo piano performances, and the third of classical symphonic music.

#### Magaloff/Chopin

The Magaloff/Chopin corpus (Flossmann et al. 2010) consists of the complete Chopin piano solo works performed by renown pianist Nikita Magaloff (1912–1992) during a series of concerts in Vienna, Austria in 1989. These performances were recorded using a Bösendorfer SE computer-monitored grand piano, and then converted into standard MIDI format.

The performances have been aligned to their corresponding machine-readable musical scores in MusicXML format, which were obtained from hard-copies of the sheet music using commercial optical music recognition software^3^ and subsequent manual correction. The score-performance alignment step has also been performed semi-automatically, involving manual correction of automatic alignments.

The data set comprises more than 150 pieces and over 300,000 performed notes, adding up to almost 10 h of music. The basis function extraction on this data produces 167 basis functions.

#### Zeilinger/Beethoven

This is another corpus of classical piano performances, very similar in form and modalities to the Magaloff/Chopin corpus. It consists of the performances of 31 different movements from 9 different Beethoven Sonatas by Austrian concert pianist Clemens Zeilinger, recorded under studio conditions at the Anton-Bruckner University in Linz (Austria), on January 3–5, 2013. The pieces were performed on a Bösendorfer CEUS 290 computer-monitored grand piano, and converted to standard MIDI format. Further preparation of the data, such as the production of machine-readable scores, and score to performance alignment were done in the same way as for the Magaloff/Chopin corpus. This data set comprises over 70,000 performed notes, adding up to just over 3 h of music. The basis function extraction on this data produces 163 basis functions.

One of the unique properties of both the Zeilinger/Beethoven and the Magaloff/Chopin corpora is that the timing and hammer velocities of each performed note have been recorded with precise measuring equipment directly in the piano, and are not based on manual annotation of audio-recordings.

#### RCO/Symphonic

The RCO/Symphonic corpus consists of symphonies from the classic and romantic period. It contains recorded performances (audio), machine-readable representations of the musical score (MusicXML) and automatically produced (using the method described in Grachten et al. 2013), manually corrected alignments between score and performance, for each of the symphonies. The manual corrections were made either at bar or at beat level (depending on the tempo of the piece), and subsequently, the performance was re-aligned automatically, using the corrected positions as anchors.

The pieces were performed by the Royal Concertgebouw Orchestra, conducted by either Iván Fischer or Mariss Jansons, at the Royal Concertgebouw in Amsterdam, the Netherlands. The corpus amounts to a total of 20 movements from five pieces, listed in Table 1. The corresponding performances sum up to a total length of over 4, 5 h of music. From the 20 scores a total of 53,816 note onsets (post merging and fusion), and 1420 basis functions were extracted.^4^

**Table 1:** Musical material contained in the RCO/Symphonic corpus

Composer	Piece	Movements	Conductor
Beethoven	Symphony no. 5 in C-Min. (op. 67)	1, 2, 3, 4	Fischer
Beethoven	Symphony no. 6 in F-Maj. (op. 68)	1, 2, 3, 4, 5	Fischer
Beethoven	Symphony no. 9 in D-Min. (op. 125)	1, 2, 3, 4	Fischer
Mahler	Symphony no. 4 in G-Maj.	1, 2, 3, 4	Jansons
Bruckner	Symphony no. 9 in D-Min. (WAB 109)	1, 2, 3	Jansons

The loudness of the recordings was computed using the EBU-R 128 loudness measure (EBU-R-128 2011) which is the recommended way of comparing loudness levels of audio content in the broadcasting industry. This measure takes into account human perception, particularly the fact that signals of equal power but different frequency content are not perceived as being equally loud. To obtain instantaneous loudness values, we compute the measure on consecutive blocks of audio, using a block size and hop size of 1024 samples, using a 44,100 Hz samplerate. Through the score-performance alignment, the resulting loudness curve is indexed by musical time (such that we know the instantaneous loudness of the recording at, say, the second beat of measure 64 in the piece), and is thus associated to the basis function representation of the piece.

### Model training

The LBM models were trained using LSMR, an iterative algorithm for solving sparse LS problems (Fong and Saunders 2011).

All NBM and RNBM models were trained using RMSProp (Dauphin et al. 2015), a mini batch variant of stochastic gradient descent that adaptively updates the learning rate by dividing the gradient by an average of its recent magnitude. In order to avoid overfitting, $l_2$-norm weight regularization, dropout and early stopping were used. Regularization of the $l_2$-norm encourages parameter values to shrink towards zero unless supported by the data. In each run of the fivefold cross-validation, 80% of the training data was used for updating the parameters and 20% was used as validation set. Early stopping was performed by monitoring the loss function on the validation set. Dropout prevents overfitting and provides a way of approximately combining different neural networks efficiently by randomly removing units in the network, along with all its incoming and outgoing connections (Srivastava et al. 2014).

The network architectures used in this study are the following:

1. *NBM* (*100, 20*) FFNN with 2 hidden layers with 100 and 20 units, respectively.
2. *RNBM* (*20rec*) vanilla bidirectional RNN with a single hidden layer with 20 units.
3. *RNBM* (*20lstm*) bidirectional LSTM with a single hidden layer with 20 units.
4. *RNBM* (*100, 20rec*) RNN consisting of a non-recurrent hidden layer with 100 units followed by a vanilla bidirectional recurrent layer with 20 units.

The hidden layers of all the above described models have *ReLU* units. We used linear output layer with a single unit for all models.

The number of layers and units in the NBM (100, 20) architecture were empirically selected from a non-exhaustive search conducted while performing preliminary experiments on joint modeling of expressive dynamics, timing and articulation. This search was performed using fivefold cross-validations on smaller subsets of the classical piano datasets (around two thirds of the pieces for each dataset) and a larger subset of basis functions.^5^ The results of these experiments lie outside the scope of this paper and, therefore, are not reported here. The RNBM (20rec) was previously used for modeling expressive timing in Grachten and Cancino Chacón (2017). We decided to use the same architecture with the more sophisticated LSTM layer, resulting in the RNBM (20lstm). Finally, the RNBM (100, 20rec) is a combination of both NBM (100, 20) and the RNBM (20rec). This architecture performs a low-dimensional encoding of the information contained in the basis functions in its first hidden layer, which then is used as an input for the recurrent layer.

All NBM and RNBM models were trained for a maximum of 2000 epochs using a learning rate of $10^{-5}$, the probability of dropout set to 0.5 and the regularization coefficient equal to $10^{-3}$. These parameters were selected empirically.

Therefore, it is important to emphasize that the results reported in this paper do not necessarily correspond to the optimal architecture for each dataset.

## Results and discussion

In this section, we present and discuss the results of the cross-validation experiments. We first present the predictive accuracies, and continue with a qualitative analysis of the results, in which we use *sensitivity analysis* methods to reveal what relations between the basis functions and the expressive dynamics the LBM and NBM models have learned. A qualitative analysis of the recurrent models is beyond the scope of this paper. We conclude the Section with some general remarks on the results.

### Predictive accuracy

Table 2 shows the predictive accuracy of the LBM and the NBM Models in the fivefold cross-validation scenario on all three corpora, and the results of the RNBM models for the RCO/Symphonic corpus. The reason for this asymmetry is that the RNBM model assumes the data to be strictly sequential. This is the case for the RCO/Symphonic data, as a result of the *fusion* operation performed on the values of the basis functions (see Sect. 3.3; Fig. 2), but not for the solo piano corpora. Since the piano recordings have MIDI velocity values per note, rather than per time position, the data instances seen by the model also correspond to notes. This means that simultaneous notes, as played in a chord, are processed sequentially, and violates the assumption that the data is strictly sequential in a temporal sense.

**Table 2:** Predictive accuracy for expressive dynamics in terms of explained variance ($R^2$) and Pearson’s correlation coefficient (*r*), averaged over a fivefold cross-validation on each of the three corpora

Model	Magaloff/Chopin		Zeilinger/Beethoven		RCO/Symphonic	
	$R^2$	*r*	$R^2$	*r*	$R^2$	*r*
LBM	0.171	0.470	0.197	0.562	$-$0.352	0.312
NBM (100, 20)	0.195	0.478	0.266	0.568	0.242	0.528
RNBM (20rec)					0.205	0.518
RNBM (20lstm)					0.271	0.590
RNBM (100, 20rec)					0.282	0.609

Due to the structure of the data, the RNBM models cannot be directly used to model expressive dynamics in the Magaloff/Chopin and Zeilinger/Beethoven corpora (see text)

The $$R^2$$ values in Table 2 quantify the proportion of variance in the target data that is explained by the model. This quantity is defined as $$R^2 = 1 - SS _{err}/SS_{y}$$, where $$ SS _{err}$$ is the sum of squared errors between the predictions and the target, and $$ SS _{y}$$ is the total sum of squares of the target. Pearson’s correlation coefficient (*r*) expresses how strongly predictions and target are linearly correlated.

Both measures show a consistent improvement of the NBM model over the LBM model, in particular $R^2$. This shows that the theoretical benefits of non-linear modeling (non-linear transformations of the input, and interactions between inputs) have practical value in the context of modeling expressive dynamics.

Further improvements can be made by modeling temporal dependencies (as in the RNBM models), but the results also show that the model needs a significant capacity in order to capture the relationship between the basis functions and expressive dynamics. In particular, RNBM (20rec) containing only 20 recurrent units performs worse than the non-recurrent NBM (100, 20) with two hidden layers of 100 and 20 units, respectively. RNBM (20lstm) *does* perform substantially better, but it should be noted that the LSTM units are composite structures themselves, with additional parameters. The best observed results have been produced by the RNBM (100, 20rec).

Prior work based on the Magaloff/Chopin data has revealed that a major part of the variance explained by the LBM is accounted for by the basis functions that represent dynamic markings and pitch, respectively, whereas other basis functions had very little effect on the predictive accuracy of the model (Grachten and Widmer 2012). To gain a better insight into the role that different basis functions play in each of the models, the learned models must be studied in more detail. For the LBM this is straight-forward: each of the basis functions is linearly related to the target using a single weight. Therefore, the magnitude of each weight is a direct measure of the impact of its corresponding basis function on the target. In a non-linear model such as the NBM, the weights of the model cannot be interpreted in such a straight-forward way. To accommodate for this, we use more generic *sensitivity analysis* methods to investigate the behavior of computational models.

### Variance-based sensitivity analysis

In order to account for the effects of the different basis functions, a *variance based sensitivity analysis* (Saltelli et al. 2010) was performed on the trained LBM and NBM models. In this sensitivity analysis, the model output *y* is treated as a function of the input basis functions $\varvec{\varphi }$ given the model parameters $\mathbf {w}$. The sensitivity of *y* is explained through a decomposition of its variance into terms depending on the input basis functions and their interactions with each other. The *first order sensitivity coefficient* $S_{1_i}$ measures the individual linear (additive) effect of the *i*th basis function $\varphi _i$ in the model output. On the other hand, the *total effect index* $S_{T_i}$ accounts for the additive effect *plus* all higher order effects of $\varphi _i$, including its interactions with the rest of the basis functions. These sensitivity measures are given respectively by

7 $$\begin{aligned} S_{1_i} = \frac{V_{\varphi _i}(\mathbb {E}_{\varvec{\varphi }\setminus \varphi _i}\left( y \mid \varphi _i)\right) }{V(y)}\quad \text{ and }&\quad S_{T_i} = \frac{\mathbb {E}_{\varvec{\varphi }\setminus \varphi _i} \left( V_{\varphi _i} ( y \mid \varvec{\varphi }_i)\right) }{V(y)}, \end{aligned}$$

where $$V_{\varphi _i}$$ is the variance with respect to the *i*th basis function, $$\mathbb {E}_{\varvec{\varphi }\setminus \varphi _i}$$ is the expected value with respect to all basis functions but $$\varphi _i$$ and *V*(*y*) is the total variance of *y*. From these definitions it is possible to show that $$\sum _i S_{1_i} = 1$$ and $$\sum _{i}S_{T_i}\ge 1$$. Furthermore, it can be shown that for a model whose output depends linearly on its inputs, as is the case with LBMs, both $$S_{1_i}$$ and $$ S_{T_i}$$ are equal.

Both $$S_{1_i}$$ and $$ S_{T_i}$$ are estimated using a quasi-Monte Carlo method proposed by Saltelli et al. (2010). This method generates a pseudo random (low-discrepancy) sequence of samples to estimate the expected values and variances in the above equations.

Table 3 lists the basis functions that contribute the most to the variance of the model, ordered according to $S_{1}$ for the LBM models trained on Magaloff/Chopin and Zeilinger/Beethoven, respectively. The columns labeled *active* specify the percentage of instances where a basis function is non-zero. This is relevant, since a high sensitivity to basis functions that are only very rarely active is a sign of overfitting. For this reason, we have grayed out basis functions that are active in less than 5% of the instances.

**Table 3:** Basis functions with the largest sensitivity coefficients for the LBM models

Magaloff/Chopin			Zeilinger/Beethoven		
Basis function	Active (%)	$S_1$	Basis function	Active (%)	$S_1$
pitch	100.00	0.168			
slur decr	63.06	0.067			
***crescendo***	42.71	0.067			
***ff***	39.12	0.059	***crescendo***	26.76	0.042
duration	100.00	0.035	***ff***	37.48	0.036
			***f***	26.07	0.036
slur incr	62.13	0.033			
***f***	35.49	0.031			
			pitch	100.00	0.024
***fff***	12.86	0.023			
					
			slur incr	35.92	0.018
			duration	100.00	0.017
			slur decr	37.66	0.017
			***diminuendo***	18.08	0.017

Averages are reported over the fivefolds of the cross-validation. Dynamics markings are in bold italic. Basis functions that are non-zero for less than 5% of the instances have been grayed out

Based on the linear model evaluated on Magaloff/Chopin it was concluded in Grachten and Widmer (2012) that pitch and dynamics markings, are important factors for predicting expressive dynamics. The results reported here, with a more diverse set of basis functions, and evaluated on a second corpus that is independent in terms of both performer and composer, roughly support this conclusion, since both pitch and a few of the most prominent dynamics markings appear as (non-grayed-out) items in the lists. The finding that pitch is (positively) correlated with expressive dynamics is in accordance both with the *High Loud* phrasing rule^6^ of the KTH model (Friberg et al. 2006), and with the unsupervised feature learning approach described in Grachten and Krebs (2014).

Furthermore, the presence of the *slur incr* and *slur decr* basis functions (see Sect. 3.2) suggests that although the slur mark is strictly hint with respect to articulation, it may act as a proxy for musical grouping, which has been shown to be related to expressive dynamics (Todd 1992).

Table 4 lists the bases to which the NBM model is most sensitive. Roughly speaking, the set of most important bases for the NBM model, conveys dynamics markings, pitch, slurs, and duration, as is the case of the LBM model. A notable difference is the high sensitivity of the NBM model to both ***crescendo*** and ***diminuendo*** markings, in both corpora. A plausible explanation for this difference is that although ***crescendo***/***diminuendo*** information in relevant for predicting expressive dynamics, the target cannot be well-approximated as a linear combination of the two basis functions. Comparing the total effect index $S_T$ and the first order sensitivity coefficient $S_1$ shows that the NBM model has learned interactions involving ***diminuendo*** and ***crescendo***. Although these values only indicate that ***diminuendo*** and ***crescendo*** interact with *some* other bases, not necessarily with *each other*, in the following we show that the latter is indeed the case.

**Table 4:** Basis functions with the largest sensitivity coefficients for the NBM models

Magaloff/Chopin				Zeilinger/Beethoven			
Basis function	Active (%)	$S_1$	$S_T$	Basis function	Active (%)	$S_1$	$S_T$
pitch	100.00	0.200	0.207				
***crescendo***	42.71	0.037	0.124	***diminuendo***	18.08	0.071	0.135
***diminuendo***	41.56	0.036	0.100	duration	100.00	0.056	0.126
slur decr	63.06	0.044	0.084	***crescendo***	26.76	0.046	0.096
***ff***	39.12	0.059	0.083	***f***	26.07	0.083	0.095
***f***	35.49	0.051	0.073	slur incr	35.92	0.046	0.080
slur incr	62.13	0.062	0.073	***ff***	37.48	0.052	0.068
duration	100.00	0.040	0.072	***p***	64.41	0.025	0.034
***fff***	12.86	0.016	0.028	slur decr	37.66	0.020	0.033
***pp***	23.18	0.006	0.020	***pp***	40.07	0.029	0.032
				pitch	100.00	0.032	0.032
							
***mp***	5.46	0.007	0.012	ritardando	31.09	0.005	0.010
***p***	41.94	0.007	0.012				
				staccato	8.41	0.005	0.005
***ppp***	5.79	0.001	0.011				

Averages are reported over the fivefolds of the cross-validation. Dynamics markings are in bold italic. Basis functions that are non-zero for less than 5% of the instances have been grayed out

**Fig. 5:**
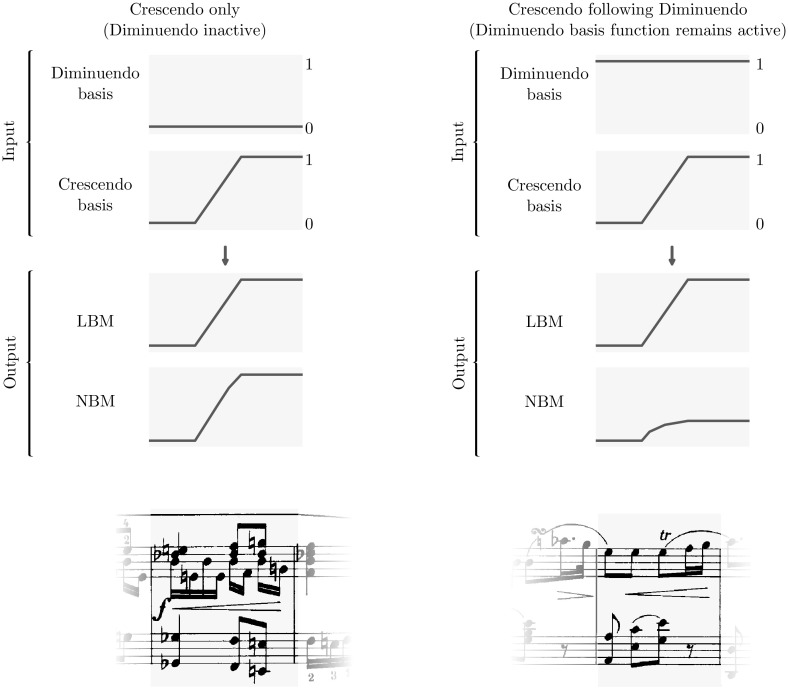
Example of the effect of the interaction of *crescendo* after a *diminuendo* for both LBM and NBM models

Figure 5 shows how both the LBM and the NBM model behave in two different scenarios concerning the occurrence of a ***crescendo***. For each scenario, a score fragment is shown (taken from the Magaloff/Chopin corpus), that exemplifies the corresponding scenario. The left half of the figure shows the scenario where a ***crescendo*** occurs (indicated by the ramp function in the ***crescendo*** input) without the interference of a ***diminuendo*** (the ***diminuendo*** input is zero). The two graphs below the inputs depict the output of the LBM and NBM models, respectively, as a response to these inputs. Apart from a slight non-linearity in the response of the NBM, note that the magnitude of the responses of both model is virtually equal.^7^

In the same way, the right half of the figure shows the response of the models to a ***crescendo*** when preceded by a ***diminuendo***. Note that the basis function encodes the ***diminuendo*** by a ramp from 0 to 1 over the range of the wedge sign, and stays at 1 until the next constant loudness annotation,^8^ such that over the range of the ***crescendo*** ramp shown in the plot, the ***diminuendo*** basis function is constant at 1.

This is a common situation, as depicted in the musical score fragment, where the musical flow requires a brief (but not sudden) decrease in loudness. Note how the response of the NBM model to the ***crescendo*** in this case is much reduced, and also smoother. The response of the LBM model, which cannot respond to interactions between inputs, is equal to its response in the first scenario.

### General discussion

The experiments show that the BM models can be used to model expressive dynamics both for different combinations of composers and performers in piano music, and for orchestral music of different composers. A question that has not been explicitly addressed in the experiments is to what degree a model trained on one combination of composer/performer is an accurate model of expressive dynamics in another combination of composer/performer. Although this question is hard to answer in general, it is possible to make some general observations. First of all, along with musical style, also performance practice has evolved over the centuries. For example, a keyboard piece from the Baroque period is typically performed very differently than piano music from the Romantic period. Models trained on one musical style should therefore not be expected to generalize to other styles. Within a specific musical style, expressive styles can still vary substantially from one performer to the other. As mentioned in Sect. 4.5 however, there are substantial commonalities in the expressive dynamics across performers (Repp 1992, 1994), taking the form of general performance principles. As the sensitivity analysis shows (Sect. 6.2), some of these principles are captured by the model, even if it is trained on the performances of a single performer. This suggest that at least to some extent, within a musical style the models may generalize from one performer to the other. However, beyond the search for general performance principles, the BM approach may be used to characterize the individuality of celebrated performers. Preliminary work in this direction has been presented in Grachten et al. (2017).

## Conclusions and future work

Expressive dynamics in classical music is a complex phenomenon, of which our understanding is far from complete. A variety of computational models have been proposed in the past, predominantly for classical piano music, as a way to gain a better understanding of expressive dynamics. In this paper we have extended a simple but state-of-the-art model for musical expression involving linear basis function modeling (Grachten and Widmer 2012), in order to investigate whether expressive dynamics can be more accurately modeled using non-linear methods. To that end we have carried out an extensive comparative evaluation of the linear and non-linear models, not only on different classical piano corpora, but also on a corpus of symphonic orchestra recordings.

The results show that non-linear methods allow for substantially more accurate modeling of expressive dynamics. A qualitative analysis of the trained models reveals that non-linear models effectively learn interaction-effects between aspects of the musical score that linear models cannot capture. Through this analysis we also find that the models reproduce several regularities in expressive dynamics that have been individually found or hypothesized in other literature, such as that high notes should be played louder, or that musical grouping (as expressed by slur marks) is a determining factor for expressive dynamics. Thus, the contribution of the present study is firstly that it provides evidence for these findings, which are sometimes no more than a musical intuition or conjecture, based on two independent data sets. Secondly, we have shown that a qualitative analysis of the models can lead to musically relevant insights such as the fact that a ***crescendo*** marking following a ***diminuendo*** tends to produce a less intense loudness increase than when occurring in isolation. This regularity, although musically intuitive (to some perhaps even trivial), to the best of our knowledge has not been formulated before. Thirdly, the models provide a unified framework in which regularities in expressive dynamics may be represented, as opposed to models that represent only a single aspect of the expressive performance.

Furthermore, on a corpus of classical symphonic recordings we have shown that modeling temporal dependencies, either using a standard bi-directional recurrent model or using a bi-directional LSTM model (Hochreiter and Schmidhuber 1997), leads to a further improvement of predictive accuracy. Although it is beyond the scope of the current paper, the temporal modeling is a promising avenue for further investigation in musical expression. In particular, it may allow for a more parsimonious representation of musical events in the score (such as a slur being described by two instantaneous events for its start and end, respectively, rather than ramp functions), since the model can use information from non-contiguous future and past events to make its current predictions.

Further prospective work is the combination of the current work with unsupervised feature learning of musical score representations (using Deep Learning) (Grachten and Krebs 2014; van Herwaarden et al. 2014). The benefit of this hybrid approach is that it combines information about annotations in the musical score, that are not part of the representation learning process but are definitely relevant for modeling expression, with the adaptiveness and malleability of representations learned from musical data.

As previously stated in Sect. 4.5, a model with high predictive accuracy might not necessarily render a good musical performance of a piece. Therefore, in addition to numerical evaluation of the model outputs, future evaluation will also involve listening tests. An interesting intermediate form between numerical evaluation and perceptual validation of model outputs is to define perceptually inspired objective functions. In the computer vision and image processing communities there have been several efforts in this direction, including the definition of the structural similarity index (Wang et al. 2004). For musical expression, a starting point is the perceptual model described in Honing (2006).

One of the limitations of the variants of the BMs presented in this paper is that they do not account for different expressive interpretations of the same score. Future work might involve probabilistic basis-models that can explicitly learn multi-modal predictive distributions over the target values. Such models are better suited to train on corpora consisting of multiple performances of the same piece (possibly by many different performers). A natural step in this direction would be the use of Mixture Density Networks (Bishop 2006; Graves 2013), which use the output of neural networks to parameterize a probability distribution. Additionally, these models allow for joint modeling of different expressive parameters (e.g. dynamics, timing and articulation) in a more ecological fashion.

## Footnotes

1 Violin, viola, violoncello and double bass.

2 Predecessors of the current BM approach have won awards at the 2008 and 2011 editions of the Music Performance Rendering Contest (Rencon) (Hashida et al. 2008), and have been evaluated favorably in a Turing test-like concert organized as part of the 1st International Workshop on Computer and Robotic Systems for Automatic Music Performance (SAMP14) (De Poli et al. 2015; Rodà et al. 2015).

3
http://www.visiv.co.uk/.

4 A complete description of the basis functions can be found in the following technical report: http://lrn2cre8.ofai.at/expression-models/TR2016-ensemble-expression.pdf.

5 The NBM (100, 20) architecture was neither the best for the Magaloff/Chopin dataset nor for the Zeilinger/Beethoven dataset, but performed relatively well given its simplicity.

6 See Table 1 in Friberg et al. (2006) for an overview of the rules of the KTH model.

7 The scale of the output is arbitrary, but has been kept constant when plotting the outputs of both the LBM and NBM, to enable visual comparison.

8 This behavior is illustrated for the ***crescendo*** sign in Fig. 1.
